# Direct and Indirect vSLAM Fusion for Augmented Reality

**DOI:** 10.3390/jimaging7080141

**Published:** 2021-08-10

**Authors:** Mohamed Outahar, Guillaume Moreau, Jean-Marie Normand

**Affiliations:** 1 Ecole Centrale de Nantes, AAU UMR CNRS 1563, 44321 Nantes, France; Jean-Marie.Normand@ec-nantes.fr; 2 IRT Jules Verne, 44340 Bouguenais, France; 3 IMT Atlantique, Lab-STICC UMR CNRS 6285, 29238 Brest, France

**Keywords:** vSLAM, direct vSLAM, indirect vSLAM, fusion, augmented reality

## Abstract

Augmented reality (AR) is an emerging technology that is applied in many fields. One of the limitations that still prevents AR to be even more widely used relates to the accessibility of devices. Indeed, the devices currently used are usually high end, expensive glasses or mobile devices. vSLAM (visual simultaneous localization and mapping) algorithms circumvent this problem by requiring relatively cheap cameras for AR. vSLAM algorithms can be classified as direct or indirect methods based on the type of data used. Each class of algorithms works optimally on a type of scene (e.g., textured or untextured) but unfortunately with little overlap. In this work, a method is proposed to fuse a direct and an indirect methods in order to have a higher robustness and to offer the possibility for AR to move seamlessly between different types of scenes. Our method is tested on three datasets against state-of-the-art direct (LSD-SLAM), semi-direct (LCSD) and indirect (ORBSLAM2) algorithms in two different scenarios: a trajectory planning and an AR scenario where a virtual object is displayed on top of the video feed; furthermore, a similar method (LCSD SLAM) is also compared to our proposal. Results show that our fusion algorithm is generally as efficient as the best algorithm both in terms of trajectory (mean errors with respect to ground truth trajectory measurements) as well as in terms of quality of the augmentation (robustness and stability). In short, we can propose a fusion algorithm that, in our tests, takes the best of both the direct and indirect methods.

## 1. Introduction

Visual simultaneous localization and mapping (vSLAM) is a family of algorithms that offers the ability to create a 3D map of an unknown environment based on a video camera feed while simultaneously being able to localize the camera in this map/environment. This represents an essential component for robotics and AR applications, which explains the wide interest of both the research and business/industrial communities for these algorithms. Even though it is vast, the vSLAM family of algorithms can be separated into two large subgroups depending on the type of basic data used. The first type, called Direct vSLAM, uses raw pixels to accomplish the tasks of localization and mapping, an example of such a direct system is the DTAM algorithm [1]. The second type, called Indirect vSLAM, uses keypoints that represent a higher level of abstraction than raw pixels, see e.g., MonoSLAM [2].

Each subgroup (direct or indirect) has a set of advantages and disadvantages. Unfortunately, none of them can address all of vSLAM’s use cases on their own. Interestingly, to some extent, they do offer complementary performances. Indirect vSLAM is known to be precise and robust against photometric changes [3]; however, since it is based on the detection of keypoints (which rely on the existence of texture), it performs poorly in untextured scenes. On the other hand, Direct vSLAM can handle scenes with little texture information [4] but has difficulties with photometric changes in the scene such as variable lighting.

The core idea of this work is to use the two methods—direct and indirect—concurrently. Our goal is to exploit the strengths of both algorithms while avoiding their weaknesses, and to use each type (direct or indirect) based on the nature of the scene.

As an indirect vSLAM algorithm, we chose ORBSLAM2 [5], and, as a direct vSLAM we chose LSD-SLAM [6].

The layout of this paper is as follows: Section 2 deals with the most relevant state-of-the-art. Section 3 details the components (ORBSLAM2 and LSD-SLAM) of our proposed system, as well as how we fuse them. Section 4 explains the testing methods and presents their results. Section 4.4 presents the discussion and the positioning of this work in the field. Section 5 concludes the paper.

## 2. State-of-the-Art

SLAM describes the problem of mapping the environment while self-localizing in the map being built. In order to accomplish these tasks, multiple sensors can be used, including Lidar [7], inertial measurement units (IMUs) [8], cameras (RGB [6], stereo [9], RGB-D [10]) or a combination of visual and inertial sensors [11]. SLAM that only uses cameras as sensors, is usually called visual SLAM, referred to as vSLAM in this paper, and is the one discussed in this work. vSLAM uses cameras to capture images and image processing algorithms to accomplish the tasks of localization and mapping. In this work, we decided to choose RGB cameras, and not stereo or RGB-D ones, because of the wide availability of RGB cameras on devices (smartphones and tablets). Furthermore, most of these devices only have one camera. Even though multiple camera devices are currently being introduced, they still represent only a fraction of the existing hardware, hence our choice for monocular vSLAM.

vSLAM algorithms can be classified in a number of ways. Aside from the one mentioned before (direct/indirect), they can also be classified based on the technique used in vSLAM for managing the map. Two major groups are distinguished: filter-based and graph-based techniques. Filter-based techniques have dominated the early days of vSLAM [2]. Examples of such techniques are EKF-SLAM [12], which uses a non-linear Kalman filter to estimate the map and trajectory, FAST-SLAM [13], which uses multiple Kalman filters and decomposes the vSLAM problem into one localization problem and multiple landmark estimation problems. The core idea is to use a filter (e.g., a Gaussian or a particular filter) to keep track of the state variables and the landmarks/map. Given the problem of quadratic growth of computational resources with the size of the map, this technique has been gradually left aside in the last decade, even though there has been a recent resurgence in filter methods [14]. The research community steered away from filter-based techniques and focused on the now widely used graph formulation. The graph-based formulation uses keyframes as nodes that are connected through poses. One of the first systems to introduce graph formulation is PTAM [15]. Since PTAM, most vSLAM algorithms use the graph formulation, including ORBSLAM2 [5], LSD-SLAM [6] and OpenVSLAM [16].

Even though this classification can be useful, the more prominent and useful type of classification of vSLAM algorithms, in our case, is based on the input data, which distinguishes between direct and indirect vSLAM. This classification is more useful because it can be directly translated into the “type” of environment where each class performs better, both in terms of robustness and precision. Indeed, direct methods work better in textureless environments with consistent lighting conditions while indirect methods perform best on textured scenes with dynamic lighting. The two subgroups are detailed below.

### 2.1. Indirect vSLAM

Indirect vSLAM relies on keypoints to track the camera and build the map. One of the first vSLAM techniques (i.e., using cameras as primary sensor in SLAM) was MonoSLAM [2], which relies on a Kalman filter, using keypoints for both tracking and mapping. Another important system is PTAM [15], this algorithm is designed for AR applications in small workspaces. PTAM was the first to propose the use of two parallel threads to run localization and mapping concurrently and not sequentially. Furthermore PTAM has introduced the use of keyframes. Keyframes are frames that are used for mapping (unlike regular frames, which are only used for tracking). Keyframes have become a standard in state-of-the-art algorithms. PTAM has introduced new advances; however, it did so without tackling the large scale mapping problem. Many algorithms have proposed solutions to address it, including FAB-MAP [17], that introduced the concept of Bag-of-Words [18] to be used for loop closing and thus handling a large scale map. Another notable work is iSAM [19], which offered a representation of the SLAM problem in the form of a graph. This formulation estimates the full trajectory and the map using all the measurements, which improves the consistency of the global map. This formulation describes the estimation of trajectory and the map as a least square problem, this reduces the computational resources needed for the tasks. Building on existing work, ORBSLAM [20] and ORBSLAM2 [5] use the presented techniques (keypoint detection/description, parallel threads, keyframes, graph formulation) to build a full vSLAM, with localization, mapping, loop closing and relocalization (i.e., the ability to relocalize the tracked object in the map after the algorithm becomes lost). ORBSLAM2 is explained in detail in Section 3.1. Other vSLAM systems are also worth mentioning: OpenVSLAM [16] is a framework that offers a highly usable and extensible indirect vSLAM with all the modules needed: keypoint detection and matching, pose optimization, keyframe processing and local and global mapping and optimization. Furthermore OpenVSLAM offers its components as separate and having application programming interfaces (APIs) to make it easier for re-usability and extensibility. OpenVSLAM is also compatible with various camera models and capable of saving and loading maps. RKSLAM [21] introduced a multi-homography based keypoint tracking as well as improvements to the local map optimization, which leads to a better performance for AR applications. With all the algorithms and the improvements proposed, indirect vSLAM, even though offering great robustness and precision, still faces major challenges when dealing with low textured scenes, due to the need for keypoints. Indeed, keypoints are detected by exploiting the intensity variations between close pixels [22], which do not exist in textureless scenes.

### 2.2. Direct vSLAM

Using raw pixels directly for tracking and mapping is a relatively new concept, with the first real-time system being introduced in 2011. DTAM [1] is one of the first direct vSLAM algorithms that uses every pixel in the image to build an estimated depth map for a (supposed static) scene. Tracking is achieved through image alignment (see Section 3.2). Another one of the earlier algorithms proposed is SVO [23], a real-time semi-direct visual odometry (VO) system. VO is similar to vSLAM but without the global mapping component. SVO uses a similar dense image alignment technique for tracking and local mapping. The same authors built on SVO to propose LSD-SLAM [6], which develops VO to full vSLAM by adding the global mapping component. LSD-SLAM offers a direct vSLAM tracking and mapping with global mapping and relocalization capabilities. LSD-SLAM is explained in details in Section 3.2.

Direct vSLAM algorithms are less prevalent since they address the smaller subset of scenes that tend to be textureless. Furthermore, direct vSLAM algorithms use photometric alignment, which means a much larger subset of pixels are used for tracking and mapping thus requiring more computational resources.

### 2.3. Other Types of vSLAM

Recently, deep learning (DL) has had a major impact on the image processing research community. This did not translate into vSLAM directly for theoretical reasons, as shown in [24], where the authors develop a theoretical model for absolute pose regression and test it to show the under-performance of convolutional neural networks (CNNs) in this task. To the best of our knowledge, there is no end-to-end vSLAM DL-based system. Deep learning, however, is used in modules in vSLAM’s broader pipeline to accomplish well defined, specialized tasks. The tasks can be: a single image pose regression [25], detection of keypoints [26], segmentation [27], object detection [28], object classification [29], relocalization [30] and visual–inertial fusion [31].

Other works have proposed, as we do here, a hybrid direct and indirect vSLAM, notably loosely coupled semi-direct monocular SLAM (LCSD) [32]. In this work, the authors implemented a system where the local mapping and tracking is achieved through the use of a direct vSLAM algorithm (DSO [4]). The data resulting from the local mapping and tracking are then exploited by the indirect vSLAM (ORBSLAM2 [5]) for back-end optimization and loop closure. The system proposes to locally run direct tracking and mapping, and refine the built map and trajectory by running a global indirect vSLAM (notably for loop closing and pose refinement). This is different from the proposed work, in that the indirect module is limited to back-end optimization and therefore cannot locally track nor build a map. Given the similarity of the methods, LCSD has been tested and compared to the proposed system, see Section 4.

The main difference between our proposal and the state-of-the-art is: the implementation of a fusion of the initialization and tracking modules, and a rigorous evaluation on multiple datasets. The system and its sub-modules are presented in details in the next section.

## 3. Materials and Methods

The basic idea is to fuse a direct and an indirect vSLAM algorithms in order to improve the overall performance and to have a more robust method. The choice of which algorithm to represent direct and indirect vSLAM was made based on both the characteristics and performance of each algorithm. Throughout the field of monocular indirect vSLAM, ORBSLAM2 is considered a reference algorithm because it is one of the first complete vSLAM algorithms, given it has the capability of tracking, global mapping and loop closing; it also offers state-of-the-art performance [3]. Considering direct algorithms, which is a less developed field, LSD-SLAM remains a leading direct algorithm in performance [33], as well as being a complete vSLAM algorithm.

### 3.1. ORBSLAM2

We chose ORBSLAM2 as the indirect algorithm in our fusion because: (i) it embeds all complementary modules (loop closing, relocalization), and (ii) the algorithm has been considered by the research community as a standard benchmark algorithm against which most other algorithms are compared, see e.g., [3,21]. In this section, the ORBSLAM2 [5] algorithm is explained in details based on the additional descriptions from [3,34].

**Figure 1:**
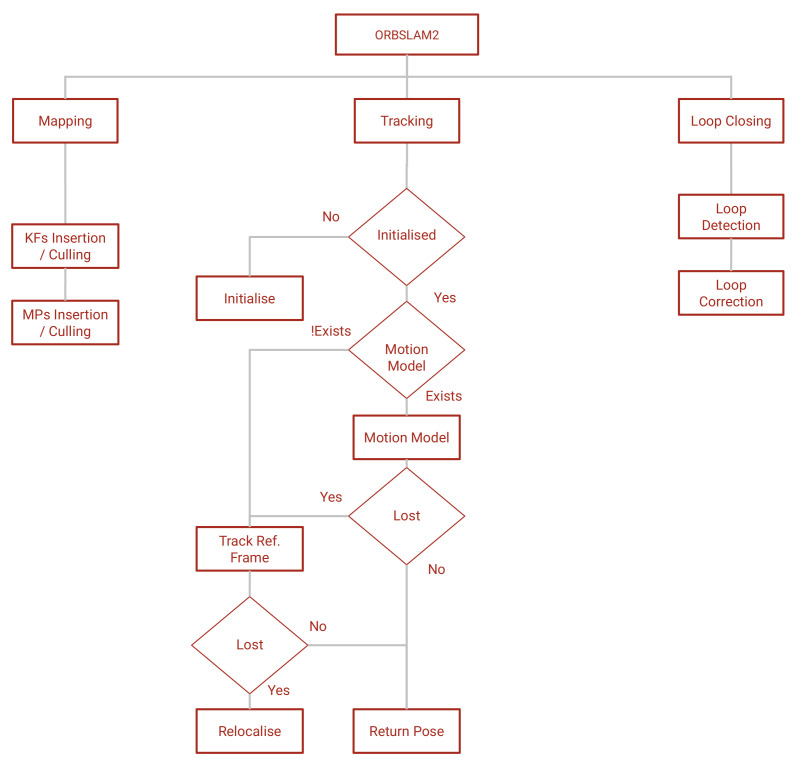
The architecture of ORBSLAM2 [5]. “KFs” means keyframes, “MPs” map points and “ref” means reference.

The algorithm runs three threads in parallel: tracking, mapping and loop closing. A diagram of the general architecture of the algorithm is presented in Figure 1.

A feature extraction module is used by ORBSLAM2 to detect, describe and match feature points. As the name indicates ORBSLAM2 uses ORB [35] feature points for both tracking and mapping.

The algorithm starts by the detection, description and matching of keypoints. The matching (or comparison) is based on the distance and orientation differences between keypoints in different frames. The initialization starts after a set number of matches between frames has been detected. The first frame is treated as a reference frame, which the other frames are compared to for matching. Once the initialization starts, the algorithm computes a fundamental matrix and a homography at the same time. Both matrices relate corresponding points in images observing the same scene; however, the homography assumes a planar scene where the fundamental matrix does not. Using both matrices, projections of keypoints are made and reprojection errors calculated. Based on the reprojection error, either the homography or the fundamental matrix is chosen. Afterwards the matched keypoints are triangulated. The triangulated points are used to populate a new map and the matched frames are used as the first two keyframes.

#### 3.1.1. Tracking

Tracking in ORBSLAM2 is performed in two ways, either with or without a motion model. The motion model represents the previous transformation between frames, the assumption is that it is still valid, given the short physical and temporal distances between frames.

When a motion model exists, it is used to project the previous frame’s keypoints to the current one. A search around the projections is carried out. If the search fails (too few matches), the window of the search is enlarged and a second search is performed. If the second search also fails, tracking without the motion model is launched.

In the absence of a motion model, the current frame is condensed into a visual bag of words and compared with the current keyframe. If the search fails to reach a number of matches (threshold fixed by the user), the relocalization module is launched.

In both cases, the algorithm performs two optimizations of the estimated pose, the first with all matched points. During the first optimization some matches are discarded as outliers. The second optimization is performed without the remaining matches. At the end of the cycle, the tracking module has to decide whether the frame should become a keyframe. The decision is made based on three criteria: number of frames from the last relocalization, number of frames from the last keyframe, number of keypoints in the current frame and difference with respect to the previous keyframe.

#### 3.1.2. Mapping

The mapping thread is responsible for optimizing, inserting and removing keyframes as well as map points. The module integrates the keyframes into the local map and, while doing so, deletes map points that are judged invalid. Afterwards, the map points are triangulated between the current keyframe and a set of keyframes that share the most keypoints with it. After these tasks are finished, and if there is no keyframe to insert, the thread goes on to perform a global bundle adjustment (BA).

In the mapping thread, three graphs are built continuously: the covisibility graph, which connects all keyframes; the essential graph, which is a reduced version of the covisibility graph, in that it connects only the keyframes that share the most keypoints; the last graph is called a spanning tree in which every keyframe is connected to the keyframe with which it shares the most features.

#### 3.1.3. Loop Closing

The last thread performs the loop closing: it checks whether the current scene has been mapped already in three steps: loop detection, loop confirmation and loop refinement. To detect loops, this module turns the current keyframe into a bag of visual words and compares to its neighbors in the covisibility graph. These comparisons result in scores. The minimal score among the neighboring keyframes is used as threshold to determine the candidates for loop closing among all keyframes. For each keyframe, a group of the closest connected keyframes is used to test the *consistence* of detection through time. If a group of keyframes is sufficiently consistent, it moves on to loop confirmation. Once a group of candidates keyframes has been chosen for confirmation, a matching between keypoint descriptors is carried out to close the loop. Given that loop closing aims at fixing scale drift, a similarity transformation (i.e., pose with scale information taken into account) is estimated as follows:

(1) $$M=\left(\begin{array}{cc} sR & t\\ 0 & 1 \end{array}\right)$$

with M∈Sim(3) a similarity, s∈ℜ the scale, R∈SO(3) a rotation matrix, $t\in {ℜ}^{3}$ a translation vector.

If the number of matches obtained by applying the estimated similarity is higher than a fixed threshold, then the transformation is accepted.

Once the transformation is accepted, the loop-closing module stops the mapping thread from making any changes to the covisibility graph until the loop is closed. The module starts by correcting the detected keyframe and the corresponding map points. Afterwards the correction is propagated through the essential graph by a global BA.

### 3.2. LSD-SLAM

**Figure 2:**
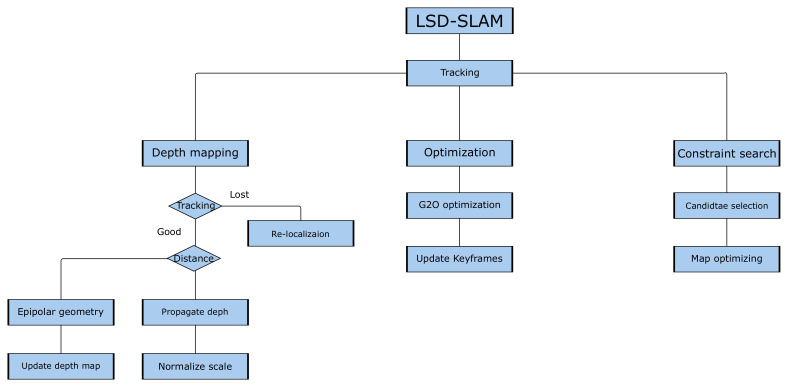
The architecture of LSD-SLAM [6]. G2O [36] is an optimization library.

LSD-SLAM is a direct vSLAM algorithm, which means that it does not use keypoints but rather pixel intensities for both localization and mapping. LSD-SLAM is built on three components: tracking, depth estimation and pose graph optimization; however, in the implementation of the algorithm, tracking is launched in the master thread, and three other threads are launched. The three threads are depth mapping, optimization and constraint search, as can be seen in Figure 2.

#### 3.2.1. Tracking

In order to estimate the pose of a frame, the tracking module aligns it with the current keyframe. This is achieved by minimizing the photometric error E(ξ). This technique is called image alignment, it estimates a pose ξ∈SE(3) by minimizing the following quantity:

(2) $$E\left(\xi \right)=\sum_{i}{\left(I_{\mathrm{ref}}\left(p_{i}\right)-I\left(\omega \left(p_{i},D_{\mathrm{ref}}\left(p_{i}\right),\xi \right)\right)\right)}^{2}$$

where ξ is the pose, $I_{\mathrm{ref}}$ is the current keyframe, $p_{i}$ are the coordinates of the selected pixel, ω is the protective wrap function that takes three parameters: $p_{i}$, $D_{\mathrm{ref}}$ the inverse depth (inverse depth is used as a workaround of infinite value problems, see [37] for details) of the pixel and ξ the pose.

In order to solve Equation (2), the variance is introduced into the optimization process as follows:

(3) $$E_{p}\left(\xi_{ji}\right)=\sum_{p\in \Omega_{D_{i}}}\|\frac{r_{p}^{2}\left(p,\xi_{ji}\right)}{\sigma_{r_{p}}^{2}\left(p,\xi_{ji}\right)}{\|}_{\delta }$$

With

(4) $$r_{p}\left(p,\xi_{ji}\right):=\left(I_{i}\left(p\right)-I_{j}\left(\omega \left(p,D_{i}\left(p\right),\xi_{ji}\right)\right)\right)$$

(5) $$\sigma_{r_{p}}^{2}\left(p,\xi_{ji}\right):=2\sigma_{I}^{2}+{\left(\frac{\partial r_{p}\left(p,\xi_{ji}\right)}{\partial D_{i}\left(p\right)}\right)}^{2}V_{i}\left(p\right)$$

where *p* are the coordinates of the selected pixel, $\xi_{ji}$ is he pose between $I_{i}$ and $I_{j}$, $I_{i}$ is the reference frame and $I_{j}$ the current frame, ω is the set of normalized pixel coordinates (they include intrinsic camera calibration), *D* is the inverse depth map, *V* is the variance map and ${\left|\cdot \right|}_{\delta }$ is the Huber norm.

The quantity described in Equation (3) is variance normalized, which means the quality of the depth estimation is taken into account.

Once the pose is estimated, the frame is processed in one of two ways depending on the distance between the current keyframe and the current frame. The distance is defined by two criteria, the estimated translation between the two frames and the number of shared points (similarly to ORBSLAM2). Based on these criteria, the module can determine whether the frame is close to the current keyframe or not. If the frame is judged to be close, as most frames are, the frame is used to update the depth map as explained in the next section; however, if the frame is judged to be too far to the current keyframe, the frame is turned into a keyframe.

#### 3.2.2. Depth Map Estimation

Frames close enough to a keyframe are used by this module to update the depth map stored in each keyframe. LSD-SLAM defines the depth of a pixel by modeling it as a Gaussian distribution $N(id,\sigma^{2})$ with mean inverse depth id and variance $\sigma^{2}$. In order to update the depth map, epipolar geometry is used, as follows.

Once the baseline and epipoles are calculated, for each pixel in the current keyframe, a visually (pixel intensity and gradients with respect to neighboring pixels) similar pixel is searched for in the current frame. Epipolar geometry constrains the search area to the epipolar line. Once the corresponding pixel is found and the depth calculated, the inverse depth distribution can be updated. The estimation is subject to two types of errors: geometric and photometric. The geometric error describes the error caused by the noise in the estimated pose. The photometric error describes the image intensity errors, this means if the image gradient is small, the pixel choice would be more prone to error.

Once all pixels are processed, a regularization iteration is performed to smooth the keyframe depth map. This is achieved by assigning to each depth value the average of the surrounding inverse depths, weighed by the inverse variance.

#### 3.2.3. Pose Graph Optimization

This step is responsible for optimizing the keyframes poses. This is a two-stage process: (i) detecting and correcting loop closures and (ii) drift correction. LSD-SLAM is a monocular vSLAM system, thus it cannot retrieve the scale of a scene, but it can keep track of it in a relative manner. LSD-SLAM does this by scaling the depth map of each keyframe to have a mean inverse depth of one and the edges between keyframes are defined as a similarities ∈ *sim*(3) (*S*E(3) plus a scale factor).

#### 3.2.4. Constraint Search

In order to close loops and find keyframes to align, a selection process is started. The module uses distance along with an appearance based process [38] in the pose graph to determine *n* candidates. For each candidate, a reciprocal tracking check is performed (tracking from candidate to current and current to candidate) and if the results are similar, the candidate is added to the global map. The convergence of *sim*(3) tracking is a limitation for direct image alignment. The algorithm overcomes this by using three methods. First, an initialization is built by choosing a set of 3D map points with correspondents in both keyframes. Second, using efficient second order minimization (ESM) [39] and lastly using a coarse-to-fine approach to find the constraints.

### 3.3. The Proposed Fusion System

**Figure 3:**
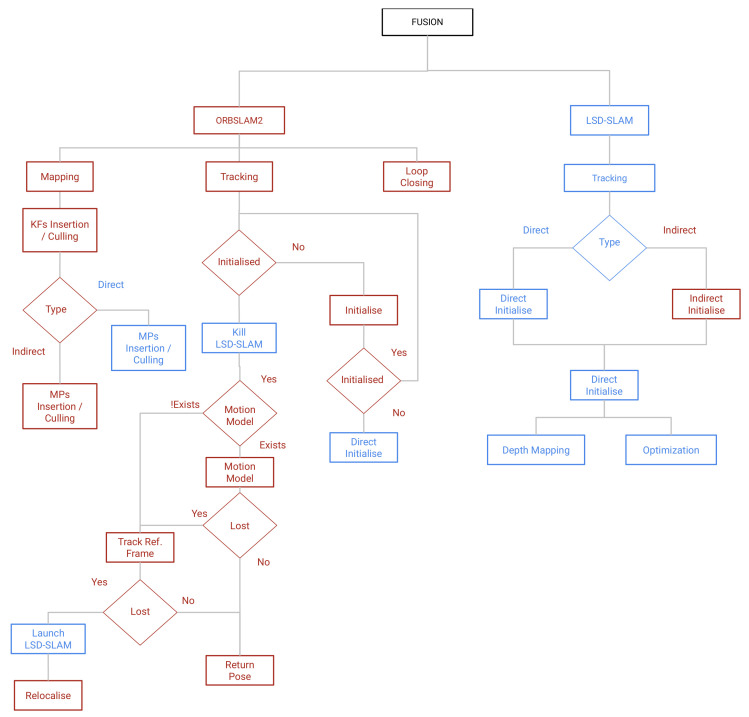
The architecture of the proposed system.“KFs” means keyframes, “MPs” map points and “ref” means reference. Blue modules are direct and red modules are indirect.

Figure 3 presents the general architecture of our system. Our proposal runs simultaneously an instance of ORBSLAM2 and one of LSD-SLAM. Each algorithm launches its own threads, namely tracking and mapping for both algorithms as well as loop closing only for ORBSLAM2.

The core idea of the proposal is to use a direct vSLAM technique to initialize and relocalize an indirect vSLAM method. With this proposal, scenes that require direct methods or indirect methods solely can both be processed. Given that indirect vSLAM performs usually better [3,5,40], we chose the indirect system (i.e., ORBSLAM2) as the *base* system.

The direct vSLAM method (LSD-SLAM) is used to initialize the indirect method. This is achieved through the extraction of the depth of the points matched between the current and the previous keyframes of the indirect method. Under the assumption that the reference frames are common between the direct and indirect methods and the median (inverse-) depth is normalized, the depth can be shared with the indirect vSLAM method. We detail how the reference frames are aligned in the following. This allows ORBSLAM2 to build the first map with two keyframes. Once this is achieved, ORBSLAM2 uses keypoint search by projecting them into the map for further tracking.

When ORBSLAM2 correctly initializes, LSD-SLAM shuts down. When ORBSLAM2 loses tracking, LSD-SLAM is re-launched with a common reference frame to carry out the tracking until ORBSLAM2 has relocalized. This strategy allows us to speedup ORBSLAM2’s initialization by using that of LSD-SLAM, which usually initializes within the first two frames. Furthermore, launching LSD-SLAM only when needed reduces the required computational resources.

Both systems are monocular so the scale is not known even though LSD-SLAM tries to estimate it. Another problem is the world frame since both systems initialize with a random reference frame. In order to fuse the two algorithms, their poses must be expressed in the same reference frame. This is achieved by identifying the transformation from one frame of reference to the other. Given the assumption that the two subsystems are estimating the same physical movement of the camera in the physical world, the estimation of this transformation is achieved by pairing the poses estimated over the same frame. As soon as both systems are successfully initialized (i.e., a first pose is computed, and an initial map is built), a transformation between both reference frames is estimated. The initial keyframes and corresponding map points are used to build the initial map.

As Figure 3 shows, ORBSLAM2 is the *base* system, and LSD-SLAM is used only when ORBSLAM2 fails. During the initialization phase, if ORBSLAM2 fails to compute a homography or a fundamental matrix, the direct initialization is carried out by assigning depth from LSD-SLAM to the initial keyframes of ORBSLAM2. Second, when ORBSLAM2 has failed to track using both the motion model and the reference frame, LSD-SLAM is initialized using the last available pose to perform tracking and mapping while ORBSLAM2 tries to relocalize.

In the next section, some practical implementation issues are presented along with the evaluation of our system.

## 4. Results

The proposed system is the integration of two large source codes, with different sub-modules and dependencies. This implementation was achieved on an Ubuntu 16.04.7 LTS system, using 16GB RAM and an Intel i7 processor. In order to visually inspect the behavior of the proposed system, the graphical viewer as well as the AR viewer of ORBSLAM2 were used.

Another notable point to raise when comparing vSLAM system is the evaluation of the trajectories, which is usually neglected in published work. In this work, we used the EVO [41] library to compare the trajectories and align them statistically.

Another point to note is the fact that, on the following sequences, the error is calculated from the first frame of the sequence. In this way the system performance is degraded proportionally to the initialization delay.

In this section, the results of the evaluation of the proposed system are presented. The evaluation is performed in two parts. The first part is the evaluation of the proposed system’s camera trajectory against those obtained from the three benchmark systems (LCSD [32], ORBSLAM2 [5] and LSD-SLAM [6]). LCSD is used as a benchmark given its similarity to the proposed method. In the compiled version of LCSD, we added input/output wrappers to process the data from the used databases, given that the developers of LCSD have made the choice in the code to only process two datasets [32].

Camera trajectories are computed on sequences belonging to three databases: TUM [42], KITTI [43] and EuRoC [44]. The TUM database represents an interior, desk space. The KITTI database represents an exterior, car view of a road. The EuRoC database represents a highly textured industrial scene.

As a metric to compare the performance of these systems, we chose to rely on the root mean squared error (RMSE). RMSE provides an idea on the typical error on a *single* point. For AR applications, the quality of the augmentations is more related to individual errors than to the mean or median errors. For that reason, we believe RMSE represents a better metric for our evaluation.

Since our final objective is to be able to use our system for AR purposes, the second part of the evaluation compares visual augmentations obtained by our proposed system with augmentations obtained via ORBSLAM2 and LSD-SLAM.

### 4.1. Trajectory Comparison

In this section, we compare camera trajectories obtained with our proposed system, LCSD, ORBSLAM2 and LSD-SLAM on three databases. The trajectories present the mean of five passes of each algorithm. In order to further clarify the evaluation process, a single scene is presented in detail.

Table 1 presents the RMSE error of the tested systems on nine sequences of the TUM database.

As can be seen from Table 1, our system has better performance on five out of nine sequences on this metric. LCSD outperforms the proposed system on *fr*2/*xyz*, this scene is a static view of a desk, this is coherent with the hypothesis of this work given that indirect methods perform better with linear movement (especially for initialization).

Table 2 presents the results of comparison between the systems on the KITTI dataset. Results show that, on this database, the proposed system performs equally to the best performing algorithm or better on four out of seven sequences. On this dataset, it can be noted that the results can be classified into three categories. The first category is where the proposed system outperforms the other three algorithms (*seq*_00, *seq*_04). The second category is where the proposed system has a equal performance to the best of the three other algorithms (*seq*_03, *seq*_06). The third and last category is the one where one (and only one) of the systems outperforms the proposed system (*seq*_01, *seq*_02 and *seq*_05).

Table 3 presents the results of comparison between the four systems on the EuRoC dataset. On this dataset, ORBSLAM2 outperforms the other three algorithms. This can be explained by the fact that the EuRoC dataset offers sequences which are highly textured and varying levels of lighting, which violates the photometric consistency hypothesis made by direct algorithms such as LSD-SLAM. It must be noted, even though our system comes in the second place, both our proposal and ORBSLAM2 are (one or two) orders of magnitude better than the two other algorithms.

The next part focuses on a single sequence. This represents one pass of the algorithms to show the consistency of the results over the sequences. This sequence is detailed in order to present other metrics than the RMSE.

**Figure 4:**
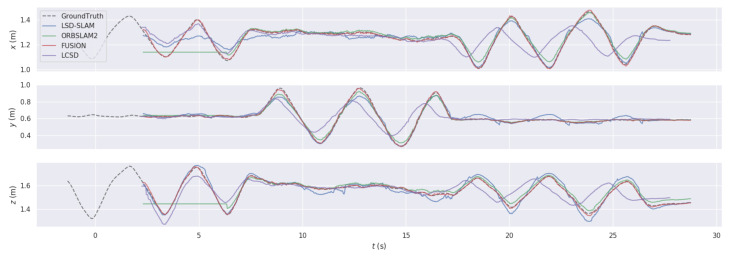
Trajectory comparisons between the proposed fusion system, LCSD, ORBSLAM2 and LSD-SLAM on the TUM sequence “*fr*1/*xyz*”.

We chose to focus on the *fr*1/*xyz* sequence of the TUM dataset. It represents a scene with both texture and untextured parts. To compare the performance of the four systems, the *x*, *y* and *z* components of the trajectory can be visualized separately, see Figure 4.

Table 4 presents the statistical characteristics of the absolute pose error (APE) given by the four systems. It shows that the proposed system has better performance than the two other algorithms, running on their own, and LCSD on every metric.

Table 4 compares the four systems in terms of seven different metrics: (i) the maximum error “max”, which describes the largest error seen throughout the trajectory with respect to the ground truth; (ii) the “mean” error, which provides an idea on accuracy of the system; (iii) “median” (the value for which half the error values is higher and the other half is lower); (iv) the minimum error (“min”), which describes the smallest error seen throughout the trajectory with respect to the ground truth; (v) “RMSE” (root mean square error); (vi) “SSE” sums of squares error; (vii) standard deviation (“std”), which describes the spread of the error, the larger this value is the less the system is precise.

As summarized in Table 4, we can see that our system is better on all metrics on this scene.

Another way to look at the results is by assuming a Gaussian error over the four trajectories, and calculating the three sigma upper limit error as can be seen in Table 5.

This means that on this sequence, using this metric, the proposed system is better by 90% than LCSD, 77% better than LSD-SLAM and 89% better than ORBSLAM2.

### 4.2. Execution Time

Our proposal is a fusion of two existing algorithms. In Section 4.1, the results show the system is generally better or comparable to the best algorithm in each type of scenes (direct or indirect). To study the added computational cost of the fusion, we chose a sequence out of every database and calculate the mean and median time per frame on our system and ORBSLAM2 as can be seen in Table 6.

Table 6 represents the processing times (and inversely frequencies) of the proposed system compared to ORBSLAM2. Two points must be noted. First, the median time is the time spent on the processing of the frame corresponding to the image in the middle of the sequence, the same method is used by ORBSLAM2. Second, in ORBSLAM2, the mean and median values are calculated based on the whole sequence (even if the system has not initialized). In the adjusted mean and median, we calculate values *only* on frames where the system is running tracking and mapping. The adjusted values are different from the non-adjusted values. This supports the hypothesis that indirect vSLAM methods have problems in initialization. We use the adjusted values for comparison with our system.

From Table 6, it can be seen that the differences between the mean of the fusion system and the adjusted mean of ORBSLAM2 is never more than 7.5%. It should also be noted that on the sequence *fr*1/*xyz* the pattern is reversed.

### 4.3. Augmentations

Since our end goal for the proposed system is to use it for AR applications, another set of evaluations involving augmentations on a set of data is presented. The augmentations are achieved by displaying a 3D cube into known coordinates of the scene (*fr*1/*xyz*). The quality of the augmentations are judged based on the stability of the virtual object with respect to the scene.

**Figure 5:**
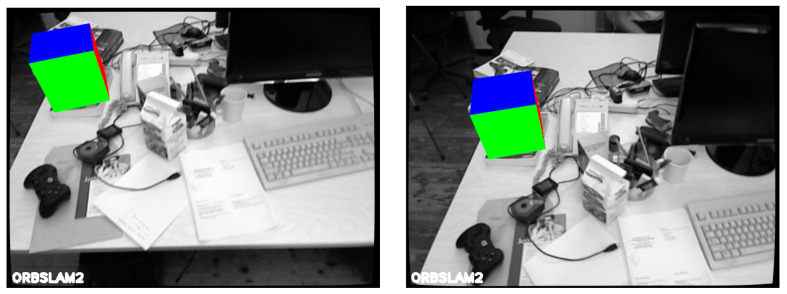
Illustration of an augmentation from our Fusion system on the TUM *fr*1/*xyz* sequence. The left and right images are taken at different moments of the augmented scene.

Figure 5 represents two frames from an augmented scene. During these two frames the proposed system runs ORBSLAM2. At the beginning of the sequence, ORBSLAM2 could not initialize, where LSD-SLAM does initialize from the second frame. Therefore the system uses LSD-SLAM to initialise ORBSLAM2, where the system adjusts the poses and map of ORBSLAM2 by the poses and map provided by LSD-SLAM as explained in Section 3.3.

As can be seen from Figure 5 the cube is relatively stable with respect to the scene. A video of the sequence can be seen here.

### 4.4. Discussion

The proposed system offers similar or better performances on the majority of sequences. The TUM dataset offers photometrically consistent scenes with textured and untextured parts—this favors direct methods. Both KITTI and EuRoC datasets are more textured and less photometrically consistent, which favors indirect methods. The proposed system has adapted to each type and performed similar or better on the three datasets. In cases where the scene has textured and untextured parts, ORBSLAM2 has difficulties initializing while LSD-SLAM and LCSD can do so more easily; However, once all the systems are initialized, then ORBSLAM2 is generally more precise. These types of scenes are more representative of the real world, which is usually a mix of textured and untextured parts.

The cases where our proposed system has a weaker performance, generally happen on scenes where one of the sub-systems (ORBSLAM2 or LSD-SLAM) fails to initialize or loses tracking. This generally happens on homogeneous (i.e., fully textured or fully untextured) sequences.

LCSD is a method quite similar to our proposal. The main difference is that LCSD uses a direct vSLAM (DSO) as a *base* algorithm. This reduces the performance on scenes that are more favorable to indirect vSLAM methods (i.e., textured and photometrically inconsistent scenes). This explains the performance of LCSD (and LSD-SLAM) on the EuRoC dataset. Furthermore, the published results of LCSD (cf. [32]) with respect to the EuRoC dataset confirms this.

The proposed system has shown that it can adapt to different types of scenes, in Section 4.2, we show that the added cost in terms of processing time is under 7.5%.

Section 4.3 presents augmentations based on the proposed system. In the augmented sequence, given that the system initialized immediately (thanks to LSD-SLAM) and the robustness of the tracking (thanks to ORBSLAM2), the virtual cube is stable from the start and does not present any jitter. This result highlights the importance of fusion of the two techniques (direct and indirect) since it allows for a seamless switch from one method to the other.

## 5. Conclusions

In this paper, we proposed a fusion system of a direct (LSD-SLAM) and an indirect (ORBSLAM2) vSLAM algorithms. Fusing different vSLAM systems does not receive a lot of attention in the community, where most attention is directed to deep learning and sensor fusion methods; however, we do believe that while these two research directions are very interesting and promising in particular for AR applications, they still present some drawbacks that fusion methods could alleviate. In particular, current DL methods cannot (yet) accomplish end-to-end vSLAM and sensor fusion methods require some specific hardware. Even if recent mobile devices are starting to embed more complex and powerful sensors (e.g., Lidars, etc.) such devices are still limited to a small market share.

We do believe monocular vSLAM still offers a large potential, especially with the fusion of direct and indirect methods. In this work, we show that fusing a state-of-the-art direct (LSD-SLAM) and indirect (ORBSLAM2) vSLAM methods can lead to better or at least equivalent performance that the best of these two methods taken individually while being usable in more realistic scenarios (i.e., combining textured and textureless environments). We also compared the proposed system against LCSD [32], which uses a direct method (DSO) for local tracking and mapping, and an indirect method for global optimization and pose refinement. We found that LCSD has a reduced performance relative to our proposal in scenes which favor indirect methods (i.e., scenes with texture and changing lighting).

As future work, we aim at developing more sophisticated fusion techniques that could increase performance and robustness and also at investigating the integration of semantic vSLAM [45] in the fusion algorithm in order to offer a wider range of AR applications (i.e., by being able to detect objects in the scene and augment them based on their semantic information).

## Figures and Tables

**Table 1:** RMSE of the four systems on sequences of the TUM database (best results in bold). N/A means the system has failed to initialize or was lost for more than 50% of the scene. The values represent the mean over five passes of each algorithm.

Sequence System	ORBSLAM2	LSD-SLAM	Fusion	LCSD
*fr*1/*xyz*	0.12390	0.05949	**0.01452**	0.07423
*fr*2/*xyz*	0.09311	0.03560	0.063873	**0.0111458**
2_360_*kidnap*	1.6157	1.66422	**1.4376**	1.58857
1_360	0.2089	0.20669	**0.2028**	N/A
1_*desk*	0.047611	0.629381	**0.04365**	0.304522
1_*desk*2	**0.7025**	0.9130	0.89436	0.8298
*fr*3_*str*_*tex*_*far*	0.46528	**0.46511**	0.580506	0.488473
*fr*3_*str*_*tex*_*near*	**0.01555**	0.72177	0.16201	0.19618
*floor*	0.5121	0.7008	**0.4334**	1.0191

**Table 2:** RMSE of the four systems on sequences of the KITTI database (best results in bold). The values represent the mean over five passes of each algorithm.

Sequence System	ORBSLAM2	LSD-SLAM	Fusion	LCSD
*seq*_00	37.8645	76.587	**31.007**	54.479
*seq*_01	408.565	379.22	279.259	**99.4138**
*seq*_02	305.26	305.425	303.352	**284.359**
*seq*_03	**152.68**	169.73	**152.68**	N/A
*seq*_04	112.781	112.973	**109.136**	N/A
*seq*_05	**27.6416**	160.466	29.1424	N/A
*seq*_06	**35.42**	137.64	**35.42**	N/A

**Table 3:** RMSE of the four systems on sequences of the EuRoC database (best results in bold). The values represent the mean over five passes of each algorithm.

Sequence System	ORBSLAM2	LSD-SLAM	Fusion	LCSD
*mav*1	**0.053**	4.162	0.071	4.157
*mav*2	**0.0325**	4.232	0.4522	4.010
*mav*3	0.0757	3.565	**0.0469**	3.496
*mav*4	**0.0661**	6.781	0.1999	6.714
*mav*5	**0.0452**	6.707	0.280	6.678
*mav*6	**0.08**	1.699	**0.08**	1.76

**Table 4:** Statistical data of the comparison between our proposed system, LCSD, ORBSLAM2 and LSD-SLAM on the sequence “*fr*1/*xyz*” in the TUM RGB-D database.

	Max	Mean	Median	Min	RMSE	SSE	std
FUSION	**0.0451**	**0.0101**	**0.0083**	**0.00048**	**0.0122**	**0.1194**	**0.0068**
LCSD	0.2988	0.1222	0.1130	0.00243	0.1416	15.943	0.0715
LSD-SLAM	0.13568	0.0495	0.0462	0.00413	0.0562	2.5085	0.0266
ORBSLAM2	0.40340	0.0552	0.0349	0.00685	0.0873	6.0390	0.0675

**Table 5:** Parameters of the Gaussian distributions of the four systems.

System	Mean	std	99% Upper Limit
Fusion	**0.0101**	**0.0068**	**0.0305**
LCSD	0.1222	0.0715	0.3367
LSD-SLAM	0.0495	0.0266	0.1293
ORBSLAM2	0.0552	0.0675	0.2577

**Table 6:** Processing times of frames in seconds is presented by subscript *t*. The inverse is presented as frequency with a subscript *f*. “Fus.” represents the results of the fusion system. All the results are a mean for five passes. For each scene, the “media” and “mean” columns represent the median and mean of ORBSLAM2 system. “Adj.” represents the adjusted times/frequencies as explained in Section 4.2.

Scene	Fus. Median	Fus. Mean	Median	Mean	Adj. Median	Adj. Mean
*fr*1/*xyz_t_*	0.0282	0.0290	0.0204	0.0227	0.0197	0.0299
*fr*1/*xyz_f_*	35.39	34.41	49.01	43.86	50.69	33.37
*seq*_06*_t_*	0.0277	0.0330	0.0274	0.0320	0.0274	0.0321
*seq*_06*_f_*	36.08	30.28	36.39	31.15	36.39	31.13
*mav*1*_t_*	0.0306	0.0336	0.0288	0.0309	0.02874	0.0311
*mav*1*_f_*	32.62	29.74	34.71	32.34	34.78	32.14

## Data Availability

The data used in this work consist of three databases, which are available at http://www.cvlibs.net/datasets/kitti/eval_odometry.php, https://vision.in.tum.de/data/datasets/rgbd-dataset and https://projects.asl.ethz.ch/datasets/doku.php?id=kmavvisualinertialdatasets. The databases were accessed on the 17 May 2020.
